# The value of AI for assessing longitudinal brain metastases treatment response

**DOI:** 10.1093/noajnl/vdae216

**Published:** 2025-01-10

**Authors:** Vincent Andrearczyk, Luis Schiappacasse, Matthieu Raccaud, Jean Bourhis, John O Prior, Michel A Cuendet, Andreas F Hottinger, Vincent Dunet, Adrien Depeursinge

**Affiliations:** Department of Nuclear Medicine and Molecular Imaging, Lausanne University Hospital (CHUV) and University of Lausanne (UNIL), Lausanne, Switzerland; Institute of Informatics, HES-SO Valais-Wallis University of Applied Sciences and Arts Western Switzerland, Sierre, Switzerland; Lundin Family Brain Tumor Research Centre, Departments of Oncology & Clinical Neurosciences, Lausanne University Hospital (CHUV) and University of Lausanne (UNIL), Lausanne, Switzerland; Department of Radiation Oncology, Lausanne University Hospital (CHUV) and University of Lausanne (UNIL), Lausanne, Switzerland; Department of Medical Radiology, Service of Diagnostic and Interventional Radiology, Neuroradiology Unit, Lausanne University Hospital (CHUV) and University of Lausanne (UNIL), Lausanne, Switzerland; Lundin Family Brain Tumor Research Centre, Departments of Oncology & Clinical Neurosciences, Lausanne University Hospital (CHUV) and University of Lausanne (UNIL), Lausanne, Switzerland; Department of Radiation Oncology, Lausanne University Hospital (CHUV) and University of Lausanne (UNIL), Lausanne, Switzerland; Lundin Family Brain Tumor Research Centre, Departments of Oncology & Clinical Neurosciences, Lausanne University Hospital (CHUV) and University of Lausanne (UNIL), Lausanne, Switzerland; Department of Nuclear Medicine and Molecular Imaging, Lausanne University Hospital (CHUV) and University of Lausanne (UNIL), Lausanne, Switzerland; Lundin Family Brain Tumor Research Centre, Departments of Oncology & Clinical Neurosciences, Lausanne University Hospital (CHUV) and University of Lausanne (UNIL), Lausanne, Switzerland; Department of Oncology, Lausanne University Hospital (CHUV) and University of Lausanne (UNIL), Lausanne, Switzerland; Lundin Family Brain Tumor Research Centre, Departments of Oncology & Clinical Neurosciences, Lausanne University Hospital (CHUV) and University of Lausanne (UNIL), Lausanne, Switzerland; Department of Oncology, Lausanne University Hospital (CHUV) and University of Lausanne (UNIL), Lausanne, Switzerland; Lundin Family Brain Tumor Research Centre, Departments of Oncology & Clinical Neurosciences, Lausanne University Hospital (CHUV) and University of Lausanne (UNIL), Lausanne, Switzerland; Department of Medical Radiology, Service of Diagnostic and Interventional Radiology, Neuroradiology Unit, Lausanne University Hospital (CHUV) and University of Lausanne (UNIL), Lausanne, Switzerland; Lundin Family Brain Tumor Research Centre, Departments of Oncology & Clinical Neurosciences, Lausanne University Hospital (CHUV) and University of Lausanne (UNIL), Lausanne, Switzerland; Department of Nuclear Medicine and Molecular Imaging, Lausanne University Hospital (CHUV) and University of Lausanne (UNIL), Lausanne, Switzerland; Institute of Informatics, HES-SO Valais-Wallis University of Applied Sciences and Arts Western Switzerland, Sierre, Switzerland

**Keywords:** brain metastases, deep learning, response assessment

## Abstract

**Background:**

Effective follow-up of brain metastasis (BM) patients post-treatment is crucial for adapting therapies and detecting new lesions. Current guidelines (Response Assessment in Neuro-Oncology-BM) have limitations, such as patient-level assessments and arbitrary lesion selection, which may not reflect outcomes in high tumor burden cases. Accurate, reproducible, and automated response assessments can improve follow-up decisions, including (1) optimizing re-treatment timing to avoid treating responding lesions or delaying treatment of progressive ones, and (2) enhancing precision in evaluating responses during clinical trials.

**Methods:**

We compared manual and automatic (deep learning-based) lesion contouring using unidimensional and volumetric criteria. Analysis focused on (1) agreement in size and RANO-BM categories, (2) stability of measurements under scanner rotations and over time, and (3) predictability of 1-year outcomes. The study included 49 BM patients, with 184 MRI studies and 448 lesions, retrospectively assessed by radiologists.

**Results:**

Automatic contouring and volumetric criteria demonstrated superior stability (*P* < .001 for rotation; *P* < .05 over time) and better outcome predictability compared to manual methods. These approaches reduced observer variability, offering reliable and efficient response assessments. The best outcome predictability, defined as 1-year response, was achieved using automatic contours and volumetric measurements. These findings highlight the potential of automated tools to streamline clinical workflows and provide consistency across evaluators, regardless of expertise.

**Conclusion:**

Automatic BM contouring and volumetric measurements provide promising tools to improve follow-up and treatment decisions in BM management. By enhancing precision and reproducibility, these methods can streamline clinical workflows and improve the evaluation of response in trials and practice.

## Lay Summary

Brain metastases (BM) are tumors that spread to the brain from other parts of the body. Doctors usually compare MRI scans before and after treatment to track changes in the tumor. The authors of this study wanted to see if automated method that use computer models could measure and track tumor changes on MRI scans better than current manual methods. To do this, they reviewed the medical records of 49 patients with BM, looking at 184 MRI scans and 448 tumors. They found that the automated measurements were more accurate than manual ones and better predicted how well patients responded to treatment.

**Key Points**

- Response assessments based on volumetric lesion measurements are more stable than unidimensional ones.
- Automatic segmentation also results in more stability than manually contoured lesions.
- Automatic volumetric assessments are also better predictive of 1-year response.

**Importance of the Study**

This study reveals the importance of automatic lesion segmentation and volumetric size measurement for individual response assessment of brain metastases.

Automatic segmentation makes volumetric assessment possible, as manual delineation is too time-consuming in clinical routine. Automatic segmentation is also more reproducible and not affected by intra- and inter-observer variability. Besides, volumetric measurements are better suited than the longest axial diameters to evaluate the size evolution since most lesions sphericity varies. Various analyses are proposed in this article to investigate these benefits.

Employing automatic volumetric response assessments can therefore result in a better patient follow-up and personalized treatment, in particular with a more reliable and earlier assessment avoiding unnecessary additional treatments (eg, pseudo-progression mistaken for progressive disease) and, conversely, a fast management of a progressive lesion. Precise response assessment is also key for identifying positive response during Phase II trials with experimental treatments.

Brain metastases (BM) originate from cancer cells that spread to the brain from primary tumors located in other sites. Research indicates that between 10% and 40% of patients with solid tumors will develop BMs over their clinical course.^1–5^

This statistic underscores the urgency of developing tailored approaches to address the unique challenges posed by BMs. Notably, cancers with high prevalence rates such as lung, breast, and melanoma cancers exhibit a substantial propensity for developing BMs,^6^ further emphasizing the need for focused studies and interventions. Stereotactic radiosurgery (SRS) is a treatment method that allows precise irradiation of individual lesions with a minimal impact on the surrounding tissue. A frequent follow-up of patients treated by SRS is particularly important to detect the appearance of new lesions and to assess the response of the treated lesions, allowing additional treatment if required.

The standard response to treatment (Response Assessment in Neuro-Oncology BMs, RANO-BM^7^) although widely used, has several significant limitations when applied to patients with BM. One of the major issues is the somewhat arbitrary selection of target lesions. Clinicians often struggle to consistently identify which lesions to monitor, especially in patients with multiple metastases. This randomness introduces a degree of subjectivity that can affect the accuracy of treatment response assessment. In addition, the selection of the MRI slice on which these lesions are measured is also somewhat arbitrary. The current RANO criteria rely heavily on measuring the longest diameter of lesions on axial MRI slices. This method can lead to inconsistencies because the measurement can vary depending on the slice selected, which may not represent the true largest dimension of the tumor. This can lead to inaccurate assessments of tumor size and response to treatment. In addition, volumes may better approximate lesion progression than longest diameters measured in the axial plane. Also, the summation of all target lesions for treatment response does not adequately reflect clinical outcomes in patients with high tumor burden. In these cases, there may be dissociated responses where some lesions shrink while others grow or remain stable. This can lead to misleading conclusions about overall treatment efficacy. Developing robust response assessment methods is crucial to (1) improve patient follow-up, where re-treatment decisions are based on reliable local progression assessment, and (2) accurately identify positive response during clinical trials with experimental treatments.

Volumetric response assessments were shown to better predict overall survival (OS) than unidimensional measures in the Response Evaluation Criteria in Solid Tumors in lung cancer.^8^ Similarly, several works evaluated the importance of volumetric-based RANO. Gahrmann et al.^9^ reported no significant improvement of the volumetric method over the RANO criteria in terms of post-treatment prognostic markers of GlioBlastoma Multiforme tumors. Huang et al.^10^ compared 1D, 2D, and volumetric criteria for the assessment of treatment response and meningioma tumor progression. The authors found a moderate inter-observer variability for all 3 methods and a modest stronger association with OS for the volumetric criteria. For BMs, the inclusion of volume measurement as a secondary endpoint is recommended by the RANO-BM guidelines.^7^ Its difference and benefits over unidimensional measurements was studied in multiple works. The increased stability of semiautomated volume measurements over diameter was associated with reduced intra- and inter-observer variability. Ozkara et al.^11^ showed that the largest diameter of a lesion may not accurately represent its volume. Oft et al.^12^ found that a cutoff of ≥20% of volumetric response at 3 months was predictive for subsequent control.

As stated in the RANO-BM guidelines,^7^ volumetric analyses add cost and complexity to the clinical practice. To speed up the assessment, semiautomatic segmentation or size measurements are frequently used in studies and clinically.^11^ The impact of MRI-based semiautomatic size assessment of BMs on the RANO-BM evaluation revealed a lower variance for semiautomatic diameter measurements, and disagreement of response assessments compared with manual measurements for 15% of cases.^13^ Fully automatic segmentation of BMs has also gained attention with recent deep learning (DL) methods,^14,15^ showing excellent performance on different modalities including T1 MRIs. This could allow for the full exploitation of volumetric response assessment potential, as evidenced in prior studies, in routine clinical settings. Ozkara et al.^11^ used automatic segmentation for correlating diameter and volume measurement.

Cho et al.^16^ trained a DL model for BM segmentation and showed that the agreements with experts were higher for the volumetric RANO-BM than the linear one.

In this work, we further compare different methods for the evaluation of response to treatment in the follow-up of BMs treated with SRS. Manual and automatic (ie, DL) methods are compared for lesion contouring and response assessment, as well as linear and volumetric assessment methods. The analyses include inter-measurement and inter-assessment agreement, size measurement and response assessment stability to rotation and across time, and predictability of outcomes. Our study aims at revealing (1) situations where methods disagree or (2) lack robustness as well as, (3) which methods are most consistent with long-term or definitive responses at a BM-level.

## Materials and Methods

### Data

The dataset originates from a retrospective, single-center, longitudinal study at CHUV^17^ in accordance with the Declaration of Helsinki, the Swiss legal requirements and the principles of Good Clinical Practice. The protocol was approved by the Research Ethics Committee-Vaud Canton, Switzerland (No. VD-CER 2024-00100). Informed consent was obtained following this approval: living patients signed a general consent, while for deceased patients, Article 34 of the Swiss Human Research Act was invoked, in line with VD-CER guidance. The dataset comprises 184 time points from 49 patients. The inclusion criteria require patients diagnosed with BMs originating from a melanoma primary cancer, treated with SRS, and imaged with a postcontrast Magnetization Prepared RApid Gradient Echo (MPRAGE) T1-weighted MRI. Patients with meningeal metastases were excluded. Patients and treatment characteristics are summarized in Table 1.

**Table 1.** Characteristics of Patients and Treatments. [a] 19 (38.8%) Patients Received More Than One Radiosurgery Treatment

Demographics	
Gender	
Females	18 (36.7%)
Males	31 (63.3%)
Age (years)	
Average	65.78
Median	66
Standard deviation	11.96
Diagnosis	
Primary site of melanoma	
Trunk	15 (30.6%)
Lower limb	9 (18.4%)
Head & neck	7 (14.3%)
Upper limb	6 (12.2%)
Mucosal	2 (4.1%)
Choroid	1 (2%)
Unknown	9 (18.4%)
Treatments	
Technique of radiosurgery (number of treatments) [a]	
CyberKnife	48
Gamma Knife	26
Systemic treatments—Number (%) of patients receiving	
Checkpoint inhibitors	
Ipilimumab (anti-CTLA-4)	27 (55.1%)
Nivolumab (anti-PD1)	21 (42.8%)
Relatlimab (LAG-3 inhibitor)	4 (8.2%)
Oncolytic viral immunotherapy	
Talimogene laherparepvec (T-VEC)	2 (4.1%)
BRaf- and MEK-selective inhibitors	9 (18.4%)
BRAF inhibitors	
Vemurafenib	14 (28.6%)
Dabrafenib	12 (24.5%)
MEK inhibitors	
Trametinib	15 (30.6%)
Cobimetinib	3 (6.1%)
Tyrosine kinase inhibitors	
Sorafenib	3 (6.1%)
Lapatinib	1 (2%)
Pazopanib	1 (2%)
Chemotherapies	
Temozolomide	11 (22.4%)
Dacarbazine	9 (18.4%)
Carboplatin-Taxol	5 (10.2%)
Nab-Paclitaxel	3 (6.1%)
Fotemustine	2 (4.1%)

### Data Processing

Images and contours are resampled to 1 mm^3^ using third-order spline and nearest-neighbor interpolation, respectively. Pairs of consecutive images are registered with the ANTS toolbox,^18^ using affine followed by deformable transformations, optimizing the cross-correlation metric.

### Manual and Automatic Contouring, Size Measurements, and Assessments

**Figure 1.**
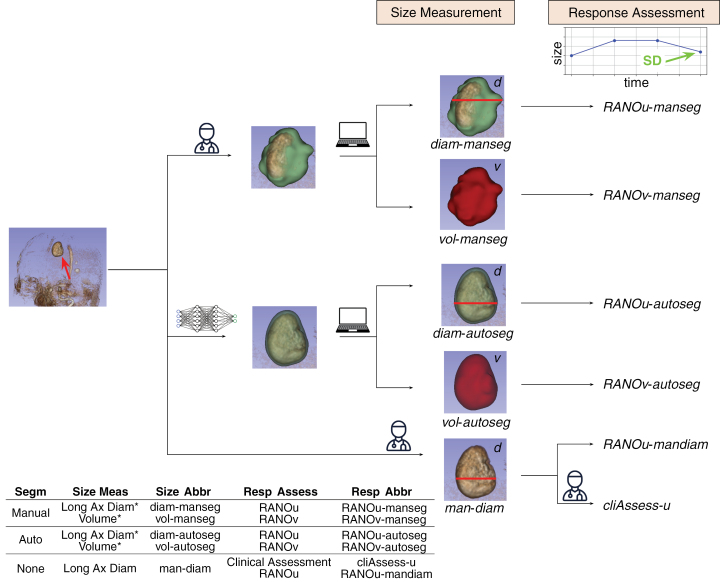
Overview sketch describing the different lesion contour, size measurements, and response assessments evaluated in this study. “^*”^ is used for measurements automatically derived from the respective lesion segmentation.

The various methods used for lesion contouring, size measurement, and response assessment evaluated in this paper are summarized in Figure 1 with the corresponding abbreviations and detailed in the following.

#### BM contouring.—

We compare two methods for BM contouring: manual and automatic. The former are manually delineated on T1

MPRAGE images by a radiologist (R1). The latter are obtained from a 3D nnUNet model^19^ described in Andrearczyk et al.^20^ The first appearance of each lesion is segmented using a standard nnUNet trained with cross-validation on 418 BMs, achieving a test dice similarity coefficient (DSC) of 0.79 and an *F*_1_-score of 0.80. The follow-ups are segmented using another nnUNet model which propagates the lesion masks from the previous time point by taking as input the T1 image together with the previous masks. This model achieves a DSC and *F*_1_-score of 0.78 and 0.88 on follow-up scans. To enable the use of all cases for the analysis, we use predictions on validation sets during 5-fold cross-validation (total of 131 time points from 36 patients) and on the separate test set (53 time points from 13 patients). All sets are separated at a patient level to avoid distinct time points from the same patients being distributed over different sets, which would result in overfitting.

#### Lesion size measurements.—

We compare 5 methods for lesion size measurements. Volume (in mm^3^) is calculated automatically from the manual and automatic contours, referred to as *vol-manseg* and *vol-autoseg*, respectively. Longest diameter is calculated automatically from the manual and automatic contours (*diam-manseg* and *diam-autoseg*), as well as manually calculated by 2 radiologists (R2) directly on the images (*man-diam*). The sets of lesions annotated by R1 and R2 are not exactly the same because some lesions, not treated by SRS, were not annotated by R2. All diameters are calculated on axial planes as performed in clinical practice. The volumes are simply calculated as the number of voxels in the respective contours since the images are resampled to 1 mm^3^.

#### Response assessments.—

A total of 6 response assessments are compared: RANO computed automatically for each lesion using the 5 lesion size measurements listed above and another assessment made by radiologists R2 (*cliAssess-u*). The latter uses the *man-diam*, which is calculated by the same radiologists R2.

The RANO-unidimensional (RANOu) is computed at the lesion level using axial longest diameter measures as follows, inspired from.^7^

- Complete response (CR): Disappearance of the lesion
- Partial response (PR): At least a 30% decrease in the longest diameter, taking as reference the baseline longest diameter
- Progressive disease (PD): At least a 20% increase in the longest diameter, taking as reference the smallest longest diameter across all time points, including the baseline if it is the smallest
- Stable disease (SD): Neither sufficient shrinkage to qualify for PR nor sufficient increase to qualify for PD

We excluded 10.9% of lesions assessed as radio-necrosis by the R2 because it is not part of RANO criteria and cannot be compared with the automatic response assessments. For the volumetric RANO (RANOv), the thresholds used for the PR and PD are 65.7% volume decrease and 72.8% volume increase, respectively. These percentages are based on an extrapolation of the diameter thresholds to a sphere.

The manual response assessment of each treated BM was performed by 2 radiologists R2. CR was considered when the lesion disappeared, PR when the lesion longest diameter decreased ≥30%, SD when the lesion longest diameter decreased *<*30% and increased *<*20% with a gado-T1/T2 match, recurrence/progression disease when the lesion longest diameter increased ≥20% with a gado-T1/T2 match, radio-necrosis (excluded for this analysis) when the lesion longest diameter increased with a gado-T1/T2 mismatch. All increase and decrease were calculated by taking as reference the previous longest diameter. Bleeding within treated lesions was also carefully scrutinized to avoid its misinterpretation as progression. Neurological symptoms, corticosteroid, or immunotherapy coadministration were not considered as we performed a lesion-based analysis.

### Analyses

#### comparisons: lesion size measurement and response assessment.—

We first evaluate a potential systematic bias in size measurement by comparing the different distributions of diameters and volumes. Significant difference in sizes is evaluated with a 2-sided Wilcoxon test.

We compute the Pearson correlation and Concordance Correlation Coefficient (CCC) between different lesion size measurement methods: (1) between diameters and between volumes to evaluate their agreement, (2) between diameter and cubic root of the volume (to account for linear correlation) to evaluate whether the slice-based nature of the manual contours negatively affects this correlation. For this second comparison of 2 correlations, we test the statistical significance of the difference with the test proposed in Pearson and Filon^21^ with a 2-sided comparison of 2 nonoverlapping correlations based on dependent groups (paired).

For comparing the response assessment methods (described in Section “Response assessments”), we compute confusion matrices to evaluate the agreement between the pairs of methods across the 4 considered response categories. We also report the percentage of agreement and Kappa agreement with and without Pabak correction.

Finally, we analyze the correlation between diameters and volumes by computing the Pearson’s correlation between longest diameters and cubic root volumes.

#### Stability of size measurements and assessments.—

We conduct this analysis with all contour types and size measurements. To simulate realistic variations in patient positioning, we rotate the 3D contours at all time points by a given range of angles (–20° to 20° with a step of 2°, resulting in 21 rotated contours per BM including the nonrotated) around the *x*-axis (and all 3 axes to simulate extreme variations in patient positioning, see Supplementary Figure 1). We measure the size (volume or longest diameter) for all rotated versions and calculate their coefficient of variation (CoV) across rotations. We take the cubic root of the volumes for the computation of the CoVs in order to ensure fair comparisons with the (unidimensional) diameters. Ideally, a simple rotation, which can happen due to a different orientation of the head, should not impact the size measurement because the lesion remains intrinsically the same. We report distributions of CoVs across all lesions. We also conduct a paired 2-sided Wilcoxon test on the differences of CoVs to compare the variation across the different contours and size measurements. The variability in size measurement due to rotation can result in changes in response assessment with RANO. To assess these variations, we also evaluate how often the rotations result in at least one change of response across the rotated lesions. For simplicity, the lesions are only rotated at the last time point, whereas the baseline and nadir are measured without rotations. We report the rate of change across all lesions and time points and perform 2-sided McNemar tests to compare method pairs.

Besides the stability to rotation, we also evaluate the stability of the size evolution in time to estimate the repeatability across time. We hypothesize that a standard evolution of size after treatment is monotonic, whereas an alternation of growth and shrinkage in consecutive follow-ups is likely related to an inaccurate size measurement. For all lesions included in this analysis, we compute the absolute Spearman correlation between the time elapsed since treatment and the lesion size. We conduct a 2-sided Wilcoxon test to evaluate the difference between the correlation coefficients of pairs of size measurement methods. Besides the monotonicity of the sizes, we also compute the rate of change of assessed response categories between pairs of consecutive follow-ups, eg, 1 change out of 2 for the baseline and 2 follow-ups [PR, CR, CR], and 2 changes out of 3 for [PR, PR, PD, PR]. We accumulate all occurrences of changes across time and lesions to compute the overall rate. We also perform McNemar tests to evaluate the significance of the rate difference between pairs of response assessment methods.

#### Predictability of outcomes.—

We investigate how the early response assessments (ie, in the first month following treatment) reflect long-term or definitive lesion outcomes. We consider the 1-year response as a final outcome, ie, first response obtained after 12 months of follow-up (ie, PD, PR, SD, or CR). We then evaluate how much time elapses after treatment until this response is observed within the first year of follow-up. Our hypothesis is that better response assessment methods will be associated with a shorter time and will have more clinical relevance. We report the distributions of times elapsed across lesions for each response assessment method. The minimum duration is the time until the first follow-up, while the maximum one is the time until the first follow-up after 1 year (ie, meaning that the 1-year response is never found in earlier follow-ups).

## Results

### Comparisons of Size Measurements and Assessments

The results of the comparison analyses, described in Section “Comparisons: lesion size measurement and response assessment,” are presented in the following.

#### Systematic bias in size measurement.—

Manually measured diameters (*man-diam*) are significantly smaller than those derived from the automatic and manual contours. Manually measured diameters (*man-diam*) (8.01 ± 0.3 mm) are significantly smaller than the ones computed from the contours: *diam-manseg* (10.64 ± 0.42 mm) and *diam-autoseg* (10.63 ± 0.42 mm). No significant difference is found between either volumes or diameters derived from the automatic and manual contours.

#### Size measurements agreements.—

We report the correlation for all lesions that were annotated by all the compared measurements. This means that we remove CRs and other nonannotated lesions from this analysis, ie, *n* = 448 for the 2 volume-based and *n* = 342 for the 3 diameter-based methods. The latter is evaluated on a smaller set because 448 − 342 = 106 lesions have manual and automatic contours from which diameters and volumes can be computed, but no manual diameter measurements, ie, *man-diam*, (computed by R2, see Section “Lesion size measurements” for more details) are available.

**Figure 2.**
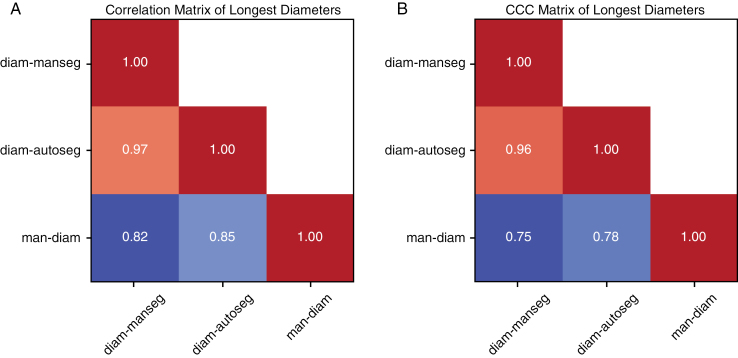
Pearson correlation matrix (A) and CCC matrix (B) of the 3 longest diameter measures (*n* = 342 lesions).

The Pearson correlation and CCC matrices of the 3 longest diameter measures are reported in Figure 2 for *n* = 342 lesions. The Pearson correlation and CCC between the 2 volume measurements (from manual and automatic contours) are 0.9902 and 0.9871, respectively for *n* = 448 lesions.

#### Response assessments agreements.—

**Figure 3.**
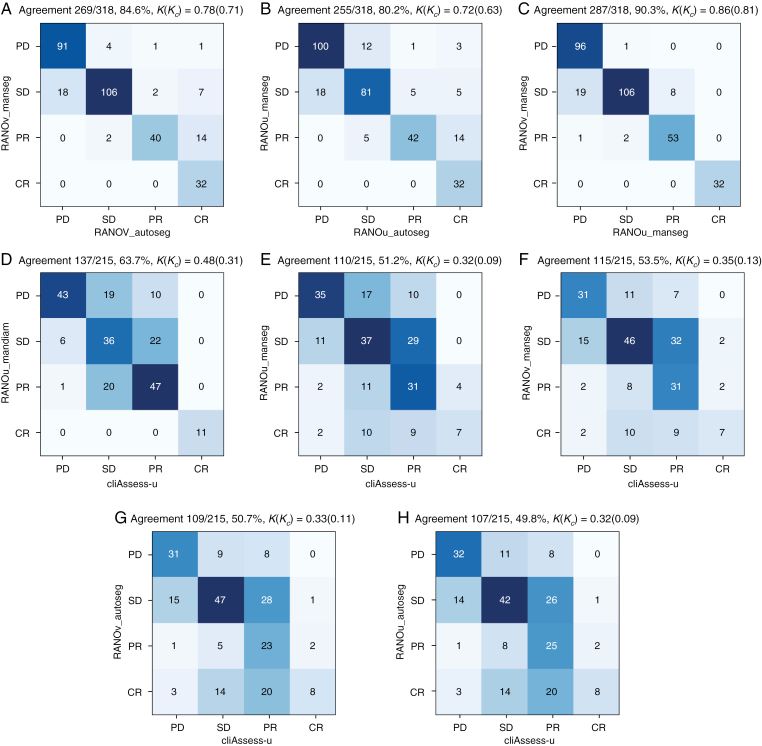
Confusion matrices comparing the agreement (A–F) between pairs of response assessment methods. The Kappa agreement score (*κ*) and Kappa with Pabak correction (*κ*_*c*_) are also reported.

Figure 3 presents the confusion matrices comparing pairs of response assessment methods. These matrices illustrate the level of agreement between different response assessment methods for various response categories (PD, SD, PR, and CR). The corresponding percentages and Kappa scores provide a summary of these agreements.

#### Diameter-volume correlation.—

To obtain a paired comparison, we use only lesions that have both manual and automatic contours (*n* = 448), similar to the size measurements agreements reported before. The Pearson’s correlation coefficient between longest diameters and cubic root volumes is 0.982 and 0.987 for the manual and automatic contours, respectively. The difference between these correlations is statistically significant (*P < *.001).

### Stability of Size Measurements and Assessments

The stability of size measurements and response assessments to rotation and across time, described in Section “Stability of size measurements and assessments,” are presented in the following.

#### Stability to rotation.—

**Figure 4.**
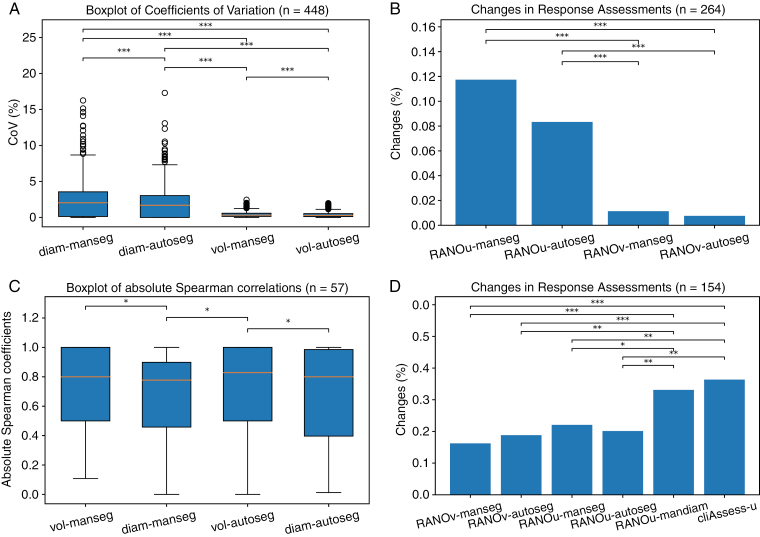
Top: stability to rotation; bottom: stability in time. (A) Boxplots of CoVs across realistic lesions rotations (single axis, see Supplementary Figure 1) for various size measurement methods and (B) corresponding rates of change in response assessments (counting at least one change of assessment across all rotations). (C) Boxplots of absolute values of Spearman correlation coefficients across lesions (hypothesis is that a nonmonotonic size evolution reflects inaccurate measurement), and (D) corresponding rates of change of response from one follow-up to the next. The significant difference in CoVs is evaluated with a 2-sided Wilcoxon test, and the difference in change rates is evaluated with a McNemar test on a contingency table made with paired occurrences. “*,” “**,” and “***” represent significance levels of 0.05, 0.01, and 0.001, respectively.

In order to obtain a paired comparison, we again use only lesions that have both manual and automatic contours (*n* = 448). The distributions of CoVs for the various size measurements (described in Section “Lesion size measurements”) are reported in Figure 4A. The stability to more extreme rotations around the 3 axes is reported in Supplementary Figure 1.

The size variation can result in different response assessments. The resulting rates of change of the different assessment methods (described in Section “Response assessments”) are illustrated in Figure 4B. The response can be assessed only in follow-up images (*n* = 264).

#### Stability in time.—

To evaluate the monotonicity of lesion size evolution in time, we use lesions with at least 3 annotated time points, ie, baseline and at least 2 follow-ups (mean and stdev of time points per lesion: 5.8 ± 2.25 in the selected ones vs 2.47 ± 2.30 for all lesions). Besides, to conduct a paired comparison, we use only lesions that have annotations across the 5 size measurement types. Figure 4C presents the distributions of Spearman correlation coefficients for the various size measurement methods and Figure 4D the corresponding rates of change of response across time.

### Predictability of Outcomes

**Figure 5.**
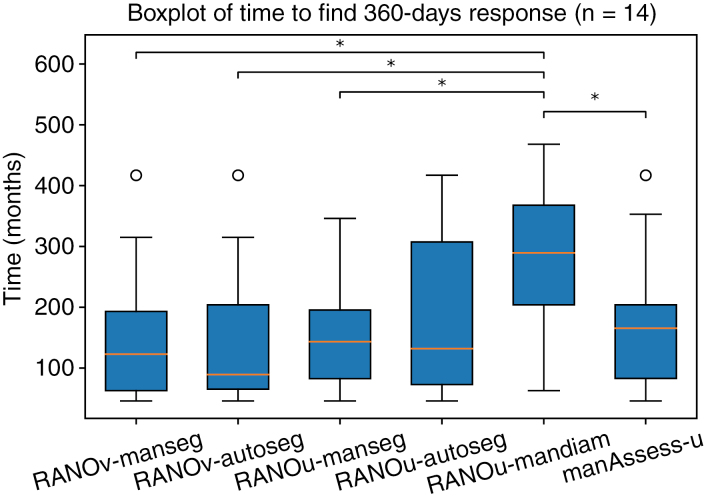
Boxplots of average time to find the 12-month response. Significance is evaluated with a 2-sided. Wilcoxon test. “*” represents a significance level of 0.05.

The results of the outcome predictability analysis, described in Section “Predictability of outcomes,” are reported in Figure 5. For this analysis, we keep only lesions with a follow-up of more than 1 year, and that have a manual assessment and manual and automatic contours. With these criteria, the number of lesions drops to only *n* = 16. The mean time in days and standard error is reported for the 6 different response assessment types.

## Discussions

High correlation and CCC are observed (Figure 2) between manual contours and automatic contours both in terms of size measurements (longest diameter and volume) and corresponding response assessments. This reflects the fact that the segmentation algorithm is trained to delineate lesions in the same way as the radiologist. This important finding suggests that automatic segmentation can be used to also automate response assessment. An important difference is found, however, between the diameters derived from the contours and the manually measured ones, where the latter were found to be significantly smaller. Pearson correlation coefficients of 0.82 and 0.85 are observed between the *man-diam* and *diam-manseg* as well as *man-diam* and *diam-autoseg*, respectively. Besides, the manual diameters are also significantly smaller than the ones computed from the contours (*diam-manseg* and *diam-autoseg*), also reflected by low CCCs. This disagreement in measurements is echoed by a low agreement (*κ* ≤ 0.35) between the RANO and manual assessments (see Figure 3E-H).

Additionally, the diameter-volume correlation is significantly higher with the automatic contours than with the manual ones (*P < *.001). Even though both correlations are high, this difference likely originates from the slice-based approach of the manual contouring resulting in coarser, sawtooth-like contours along the inferior-superior (*z*) axis.

Volumes are more stable to rotations than diameters, as illustrated by the distributions of CoVs in Figure 4A. The small variations in volume measurements are due to interpolation. Measures derived from automatic contours are also more stable than those derived from manual contours. While we could not compute the stability to rotation of the manually calculated diameters (*man-diam*) as it would require highly time-consuming additional annotations, similar trends are expected. These differences in measurement stability are also reflected in significant differences in terms of associated changes in response assessments as depicted in Figure 4B.

Similarly to the stability to rotation, the results consistently support volumes as the most stable size measurement method across time. We evaluated the Spearman correlation between time and size, where a high correlation reflects a stable monotonic evolution of the size measurement across time (Figure 4C). A significant difference is found between volumetric and diameter measurements based on either manual or automatic contours. This difference in stability is also reflected by a lower rate of response change across time of RANO based on volume measurements (Figure 4D), suggesting that current clinical practices could be optimized.

The number of lesions with a follow-up longer than 12 months is small for the analysis of outcome prediction (*n* = 14). As shown in Figure 5, all response assessment methods have a similar average elapsed time for 12-month outcome predictability (∼160 days) except for the RANO based on manually calculated diameters (270 days). In particular, the latter is longer than the manual RANO (182 days), which uses the same manually calculated diameters. This may reflect the fact that information additional to the manual diameter was used for the manual assessment (as also shown by the low agreement in the confusion matrix in Figure 2). The manual assessment seems to better predict the 1-year response, with similar scores to the RANO with automatically calculated sizes.

Volumetric assessment provides a more objective and comprehensive method for evaluating the response of BM to treatment. By measuring the volume of each lesion, this approach provides a more accurate representation of tumor burden and its changes over time. Volumetric measurements are less susceptible to the variability introduced by the selection of specific MRI slices and the inherent subjectivity of manual diameter measurements. One of the key advantages of volumetric assessment is its ability to account for heterogeneity in response among multiple metastases within the same patient. In patients with high tumor burden, volumetric assessment can differentiate between lesions that respond to treatment and those that do not, providing a more nuanced understanding of the patient’s overall response. This is particularly important in the context of modern treatment approaches, where the goal is often curative, and accurate assessment of each lesion is critical for treatment planning and adjustment.

This study suffers from limitations, including its retrospective nature and the fact that we only include BMs originating from melanoma primary cancers, with a limited sample size and follow-up (225 ± 264 days) and heterogeneous systemic treatments. Besides, the automatic contours have FNs and FPs, which are not reflected by most of the analyses presented here since we conduct the experiments (eg, stability to rotation and in time) only on lesions without CR. We also use segmentation predictions of the DL model obtained in the cross-validation. It is worth noting that the goal of the paper is not the full evaluation of the automatic segmentation model, which is reported in Andrearczyk et al.^20^ The performance of the re-segmentation algorithm remains a potential limitation for clinical adoption. Another limitation is the fact that all size measurements are not available for all lesions since some are only annotated by the manual and automatic contours and not by the manual response assessment because not treated by SRS. For some comparisons, we kept the intersection of annotated lesions to enable a paired comparison. It is also worth noting that we do not define a ground truth for the size measurement and response assessment since we compare different methods without knowing which is best. Despite the absence of ground truth, we aim to reveal the best method in terms of stability and 1-year response prediction.

Other limitations are specific to individual analyses. The stability to rotation and across time is not as such a proof of the superiority of one measurement or assessment method over another: a random yet constant size measurement or response assessment would result in perfect stability. We also assume monotonicity, of the size evolution which may not always reflect the true lesion evolution (eg, pseudo-progression and pseudo-response). Finally, the small number of lesions with long follow-up (*n* = 14) hinders solid conclusion drawing for the outcome predictability experiment.

The introduction of automated volumetric evaluation, as proposed in our study, can significantly improve the accuracy and reliability of treatment response assessment. Automated segmentation tools powered by DL algorithms can streamline the process and make it feasible for routine clinical use. These tools can consistently delineate lesion boundaries, reduce inter- and intra-observer variability, and ensure that volumetric measurements are accurate and reproducible. The implementation of volumetric evaluation and automatic segmentation has the potential to significantly change patient management. In particular, the following clinical implications are noteworthy: (1) optimized follow-up schedules, (2) improved predictive power, (3) better resource allocation, (4) improved robustness of clinical trial data.

## Conclusion

While the RANO criteria have been instrumental in standardizing response assessment in neuro-oncology, their limitations necessitate the exploration of more robust methods. Volumetric assessment represents a significant advancement, providing a more objective and detailed understanding of treatment response, particularly in patients with complex and heavily burdened metastatic disease. Our study highlights the potential of this approach to improve clinical outcomes by increasing the accuracy and reliability of BM assessment. The implementation of automatic segmentation allows the use of volumetric measurements to assess response in BMs. Although there is a moderate level of agreement between manual assessments and automated RANOs, the latter can provide clinicians with valuable, objective, and consistent information to determine the optimal treatment strategy for BMs and patient follow-up. Volumetric lesion size measurements and their corresponding automatic response assessments were found to be more stable than unidimensional ones. In addition, size measurements from automatic contours and their corresponding response scores were more stable than their counterparts based on manual contours and fully manual measurements and assessments. Similarly, automatic measurements appear to be more suitable for early detection of the final BM response. These findings support the use of (1) automatic lesion segmentation, (2) volumetric measurement, and (3) automatic response assessment to assist radiologists in their daily clinical routine for patient follow-up. In future work, a larger cohort with other primary cancers will allow us to confirm the results and investigate the prediction of future lesion response from single and multiple time points. Volumetric assessment is a significant advance that provides a more objective and detailed understanding of treatment response, particularly in patients with complex and heavily burdened metastatic disease. This approach may lead to improved clinical outcomes by increasing the accuracy and reliability of BM evaluation.

## Supplementary Material

vdae216_suppl_Supplementary_Appendix

## Data Availability

The data analyzed in this study are not publicly available as not permitted by the ethics agreement.
