# Supplementary figures and images for: The value of AI for assessing longitudinal brain metastases treatment response
Source: Neurooncol Adv. 2025 Jan 10;7(1):vdae216. doi: 10.1093/noajnl/vdae216 (PMC11786217; doi:10.1093/noajnl/vdae216)

## Slide 1
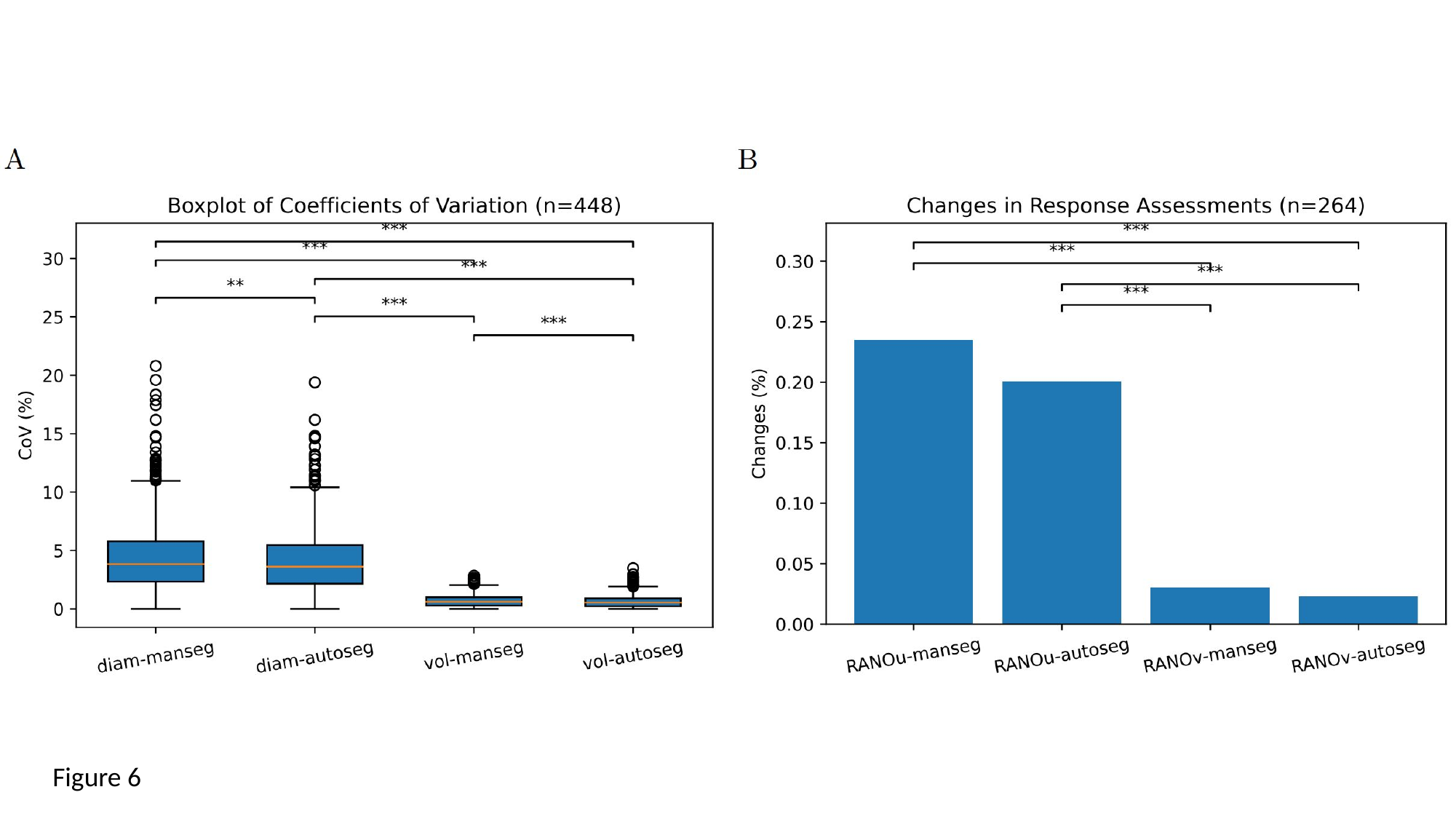

Figure 6

Supplement: vdae216_suppl_Supplementary_Appendix [file vdae216_suppl_Supplementary_Appendix.pptx]
